# Traditional medicinal plant knowledge and use by local healers in Sekoru District, Jimma Zone, Southwestern Ethiopia

**DOI:** 10.1186/1746-4269-3-24

**Published:** 2007-06-04

**Authors:** Haile Yineger, Delenasaw Yewhalaw

**Affiliations:** 1 Department of Biology, Jimma University, P.O.Box 5195, Jimma, Ethiopia

## Abstract

The knowledge and use of medicinal plant species by traditional healers was investigated in Sekoru District, Jimma Zone, Southwestern Ethiopia from December 2005 to November 2006. Traditional healers of the study area were selected randomly and interviewed with the help of translators to gather information on the knowledge and use of medicinal plants used as a remedy for human ailments in the study area. In the current study, it was reported that 27 plant species belonging to 27 genera and 18 families were commonly used to treat various human ailments. Most of these species (85.71%) were wild and harvested mainly for their leaves (64.52%). The most cited ethnomedicinal plant species was *Alysicarpus quartinianus *A. Rich., whose roots and leaves were reported by traditional healers to be crushed in fresh and applied as a lotion on the lesions of patients of *Abiato (Shererit)*. No significant correlation was observed between the age of traditional healers and the number of species reported and the indigenous knowledge transfer was found to be similar. More than one medicinal plant species were used more frequently than the use of a single species for remedy preparations. Plant parts used for remedy preparations showed significant difference with medicinal plant species abundance in the study area.

## Background

Traditional medicine has remained as the most affordable and easily accessible source of treatment in the primary healthcare system of resource poor communities and the local therapy is the only means of medical treatment for such communities.

In Ethiopia, medicinal plants have been used as traditional medicine to treat different human ailments by the local people from time immemorial. These medicinal plants are estimated to be over 700 species [1] and most of them are confined to the southwestern regions of the country [2].

There is a high expectation of enormous traditional knowledge and use of medicinal plant species in Ethiopia due to the existence of diverse cultures, languages and beliefs among the people. However, since cultural systems are dynamic [3], the skills are fragile and easily forgettable as most of the indigenous knowledge transfer in the country is based on oral transmission [4]. To our knowledge, there are no data regarding the traditional medicinal plant knowledge and use by the local communities in Sekoru District, Southwestern Ethiopia. Therefore, the current study was conducted to assess and document the indigenous knowledge and use of medicinal plant species by traditional healers to treat human ailments in the study area.

## Methods

### Study area

**Figure 1:**
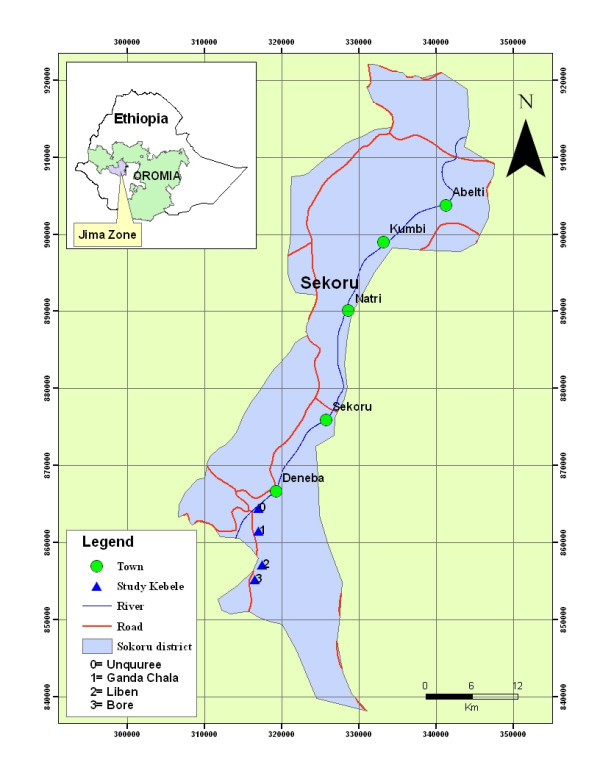
Map showing location of the study sites.

The study was conducted in four *Kebeles *(the smallest administrative units in Ethiopia) of Sekoru District, Jimma Zone, Southwestern Ethiopia from December 2005 to November 2006. The four selected *Kebeles *were Unquuree, Ganda Chala, Liben and Bore (Figure 1). These areas lie at an altitudinal range of 1693 m – 1740 m a.s.l. The study area has a dry and hot climate with a mean annual temperature of 19.2°C and annual rainfall that varies from 1300 mm – 1800 mm. Clay soil with a thin layer of humus top soil is the main soil type over the area and evergreen montane thickets and shrubs are characteristic vegetation types of the area. The socioeconomic activity of the local population is mainly mixed farming which involves both cultivation of crops and rearing of livestock. *Zea mays *L., *Sorghum bicolor *(L.) Moench and *Eragrostis tef *(Zucc.) Trotter are the major crops cultivated in the area.

### Data collection

An ethnobotanical study was conducted on four selected *Kebeles *of Sekoru District, Jimma Zone, Southwestern Ethiopia from December 2005 to November 2006. The coordinates of study *Kebeles *were recorded using a hand held GPS unit. Ethnobotanical data were collected from 13 randomly selected traditional healers using semi structured interviews and participant observations following [5]. The traditional healers involved in the study were all males and their ages ranged from 30 to 84. Most of the healers were illiterate (53.85%) and at most only able to read and write (30.77%) while few (15.38%) attended up to standard 4. Ethical clearance was sought from Jimma University Ethics Review Committee while verbal informed consent was obtained from each individual traditional healer who was participating during the study period.

Interviews were made with each traditional healer about the knowledge and use of medicinal plant species used to treat human diseases in the study area. The healers were professional practitioners who medicate the local people by using ethnomedicinal plants and their products. The interviews were facilitated with translators who were well conversant of the local language (*Oromiffa*). Data on human ailments treated, local name of plants used, growth form, degree of management (wild/cultivated), parts used, methods of preparation, route of administration and application, added values of medicinal plants, existing threats to medicinal plant species and indigenous knowledge transfer were recorded. The authors accompanied the traditional healers and translators and made field visits to observe and collect medicinal plant species reported to treat ailments. Voucher specimens of each medicinal plant species were also collected during the field visits and allotted collection numbers. The collected specimens were then dried, identified and deposited at Jimma University Regional Herbarium and at the National Herbarium, ETH (Addis Ababa University). The specimens were identified using herbarium materials, experts, and taxonomic keys in the various volumes of the Flora of Ethiopia and Eritrea [6-12].

### Data analysis

Binomial test, chi-square (X^2^) test and the Spearman rank correlation test were run in SPSS 12.0.1 to analyze ethnobotanical data. The Spearman rank correlation test was used to determine the correlation of the indigenous plant use knowledge with the age of traditional healers and their level of education whereas chi-square test was used to determine whether there was a significant variation on the abundance of traditional medicinal plant species with regard to their parts used, degree of management (wild/cultivated), marketability and added values. Moreover, binomial test was employed to evaluate whether remedies were prepared from single species, healers had good knowledge of dosages, traditional medicines had adverse effects, marketable species were more frequently used, and whether healers used to transfer their indigenous knowledge. MS Excel Spreadsheet was also utilized for drawing bar graphs and to determine proportions.

## Results

Traditional healers of the study area were found to play great roles in the primary healthcare systems of the local people as they were treating resource poor people who had little access and couldn't afford the cost for modern medications. They also reported that the local people have been seeking for their treatment even in preference to modern medications and also in connection with the community's belief that they would not get better medications for some of the diseases in modern health services. Moreover, the traditional healers indicated that they served the community best as the distribution of health services was limited in the study area.

No significant (Spearman correlation test, r = 0.140, α = 0.05, p = 0.365) correlation was observed between the age of traditional healers and the number of species reported by the healers. Moreover, the Spearman correlation test did not demonstrate significant (Spearman correlation test, r = 0.258, α = 0.05, p = 0.091) correlation between the educational level of traditional healers and the number of species reported. The response of the traditional healers with regard to their indigenous knowledge transfer was similar (binomial test, p = 0.880) in that some of them reported to transfer it to selected family members while some did not transfer it at all.

Altogether, seventeen human diseases or ailments were identified by the traditional healers of the study area. Twenty seven traditional medicinal plant species distributed among 27 genera and 18 botanical families were also cited by the traditional healers to treat those ailments (see Additional file 1). Fabaceae was the most represented family in terms of medicinal plant species diversity followed by Acanthaceae, Cucurbitaceae, Lamiaceae, Loranthaceae, Myrsinaceae and Verbenaceae.

Among the cited medicinal plant species of the study area, the majority (85.71%) were wild. Whereas 10.71% of the reported medicinal plant species were both cultivated and wild. Few species (3.57%) were indicated as cultivated. Moreover, significant (χ^2 ^= 24.080, df = 12, α = 0.05, p = 0.020) difference was observed between the degree of management (wild/cultivated) and abundance of the medicinal species in the area. The dominant growth forms among the reported medicinal plant species were shrubs (37.04%) and trees (25.93%). The proportion of the remaining growth forms was similar (7.41%).

Most of the species (64.52%) were harvested for their leaves to prepare remedies. Preparation of remedies from the roots of some (19.35%) medicinal plant species was also reported by traditional healers of the study area. Plant parts used for remedy preparations also showed significant (χ^2 ^= 70.158, df = 36, α = 0.05, p = 0.001) difference with medicinal plant species abundance in the study area. About 64.52% of the medicinal species were cited to be used in fresh form in remedy preparations. Relatively few medicinal plant species were reported to be used in dried (19.35%) and fresh or dried (16.13%) forms.

**Figure 2:**
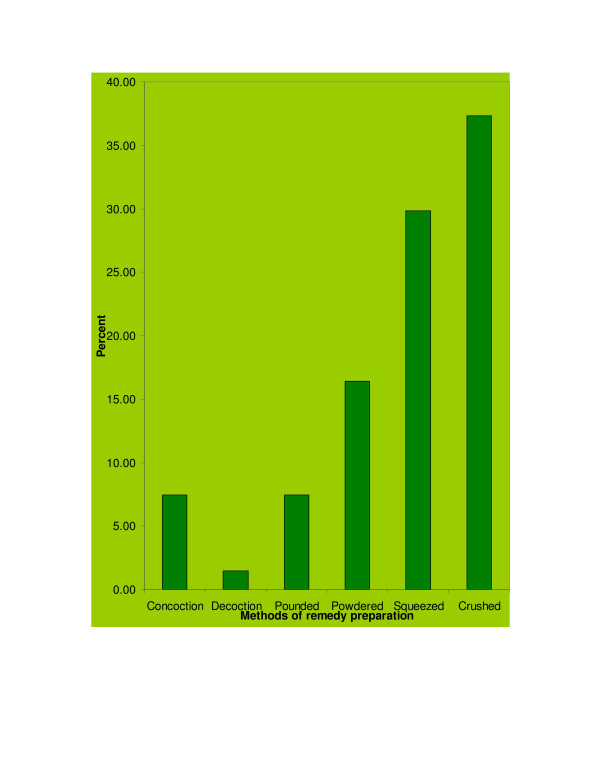
Methods used by traditional healers in remedy preparation.

The principal methods of remedy preparation were reported to be through crushing (37.31%), squeezing (29.85%) and powdering (16.42%) of the various parts of medicinal plants (Figure 2). Additives like sugar, honey, tea, coffee, edible oil and garlic were used in most (60%) of the remedy preparations. Moreover, more than one medicinal plant species were used more frequently (binomial test, p = 0.000) than the use of a single species for remedy preparations. Remedies for tumor (*Tanachaa*) were reported to be prepared by crushing fresh leaves of *Tapinanthus globiferus *(A. Rich.) Tiegh. and mixed with cold water to be administered orally. The powdered dry root of *Gloriosa superba *L. was administered orally along with tea and coffee to treat this ailment. Similarly, powdered root of *Plumbago zeylanica *L. was reported to be mixed with water and sugar and tumor patients drank half cup of the preparation for three days. Twigs of this species were also indicated to be put on the neck of patients as necklace and this was believed by traditional healers to cure the ailment. Freshly crushed roots of *G. superba *and *Clerodendrum myricoides *(Hochst) R. Br. ex Vatke were indicated to be squeezed with water and drunk against this ailment.

Healers used freshly crushed fruits of *Gardenia ternifolia *Schumach. & Thonn. to treat haemorrhoids lesions. The dried fruits of this species were alternatively powdered and directly applied over infected sites of patients of this disease.

Fresh leaves of *Calpurnia aurea *(Ait.) Benth., *Clematis hirsuta *Perr. & Guill., *Engleriana woodfordioides *(Schweinf.), *Myrica salicifolia *Hochst. ex A. Rich. and *Plectranthus rupestris *Vatke ex Baker were crushed, squeezed and a small amount of the juice was applied through auricular route for two days to treat earache (*Dhukuba Guraa*).

Juice prepared from pounded and squeezed fresh leaves of *Croton macrostachyus *Del., *Premna schimperi *Engl. and *Vernonia amygdalina *Del. were applied as a lotion on the lesions of patients of *Abiato *(*Shererit*). The remnant was also reported to be dried, powdered and applied over the lesions. Roots and leaves of *Alysicarpus quartinianus *A. Rich. were also crushed in fresh and put on the lesions of the patients.

Leaves of *A. quartinianus*, *Clausena anisata *(Willd.) Hook. f. ex Benth., *M. salicifolia*, *Myrsine africana *L. and *P. schimperi *were reported to be crushed and squeezed in fresh form with water. The juice was then indicated to be drunk in very small amount for three days to treat *Naqarsaa*.

Fresh roots of *Entada abyssinica *Steud. ex A. Rich., *Momordica foetida *Schumach. and *Oreosyce africana *Hook. f. were crushed and squeezed with water. The filtrate was reported to be given through hypodermal injection using a syringe to treat gonorrhoea (*Dhukuba Dhiraa*).

**Figure 3:**
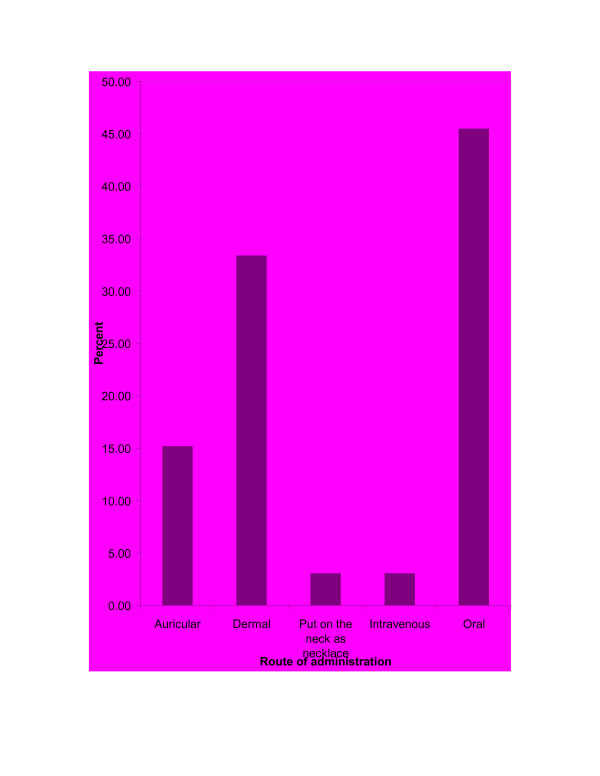
Route of traditional medicine administrations.

Remedies were reported to be administered mainly through oral (45.45%), dermal (33.33%) and auricular (15.15%) routes (Figure 3). However, knowledge of traditional healers on dosage of each remedy was poor (binomial test, p = 0.004). The absence of any adverse effects of traditional medicines after administration were also more frequently mentioned (binomial test, p = 0.008) by the traditional healers but some of the preparations were reported to have some adverse effects like vomiting and temporary inflammations on patients. Interestingly, all of the traditional healers indicated that they did not use antidotes for the adverse effects of traditional medicines.

The most cited modes of remedy applications were drinking (42.86%), topical applications (28.57%) and dropping (14.29%).

**Figure 4:**
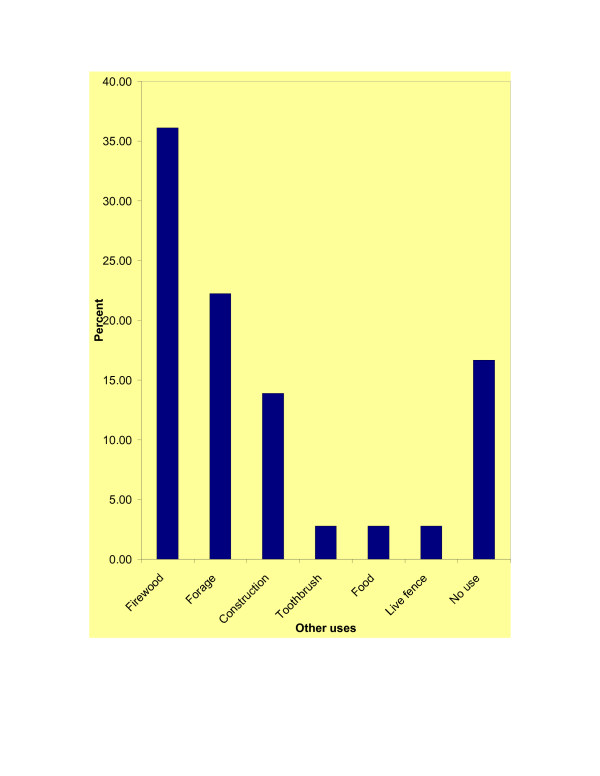
Added values of traditional medicinal plant species.

Non-marketable medicinal species were more significantly cited (binomial test, p = 0.000) by the traditional healers. The abundance of marketable and non-marketable medicinal plant species were significantly (χ^2 ^= 46.728, df = 8, α = 0.05, p = 0.000) varied. The majority of the recorded medicinal species were reported as abundant (36.67%) and rare (33.33%). Some species were also mentioned as very abundant (16.67%) and very rare (13.33%). The abundance of reported medicinal plant species also showed significant (χ^2 ^= 34.824, df = 8, α = 0.05, p = 0.000) variation with their added values. Most of the medicinal plant species were reported to have added values to the local people. The most cited uses were firewood (36.11%), forage (22.22%) and construction (13.89%). Some species (16.67%) had no uses other than their medicinal value (Figure 4).

**Figure 5:**
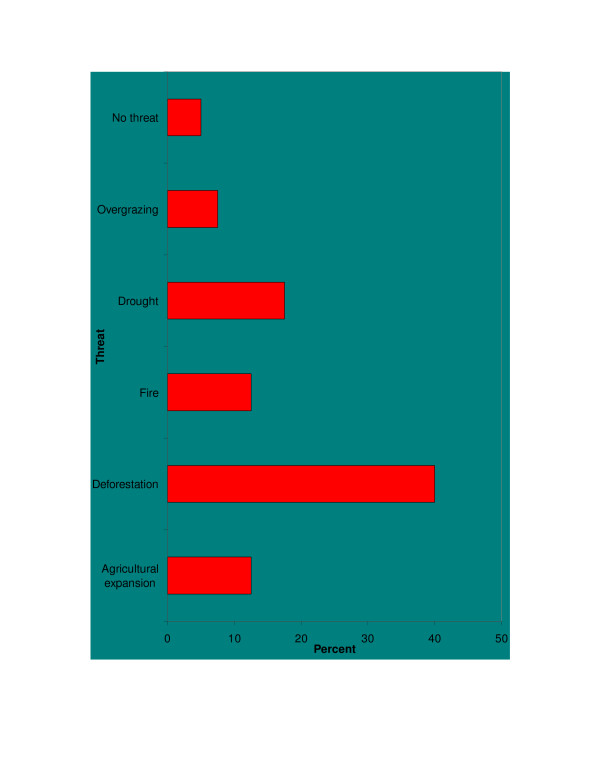
Reported threats to medicinal plant species of the study area.

The most mentioned threats to medicinal plants of the study area were deforestation (40%), drought (17.5%) agricultural expansion (12.5%) and fire (12.5%) (Figure 5).

## Discussion

The use of traditional medicinal plants to treat human ailments is not exhaustively documented in Ethiopia despite studies had been conducted in some areas of Northern [13], Northwestern [14,15], Central [16-18], Southeastern [19-21] and Southwestern [2] Ethiopia. In the current study, a total of 27 medicinal plant species in 27 genera which were used by traditional healers were recorded from four *Kebeles *of Sekoru District, Jimma Zone, Southwestern Ethiopia.

Result of this study showed that most of the medicinal plant species used were reported as wild. Similar studies conducted in Ethiopia [19,16,21], Uganda [22,23], Serbia [24] and in Northern Peru [25] indicated that most medicinal plant species used to treat human ailments were wild. This implies that the majority of plants of medical importance were not yet cultivated by traditional healers.

Shrubs and trees were the most represented growth forms for remedy preparations in the study area. This could be due to the fact that these growth forms are available in almost all seasons as they are relatively drought resistant and are not affected by seasonal variations [26].

Leaves were the most cited plant parts used by the healers for the preparation of traditional medicines. This finding is in line with the results of other ethnomedicinal studies in Africa [16,27,23] and elsewhere [28,29] who reported that leaves were the most cited plant parts used in remedy preparations.

The result of this study showed that more than one plant species were mostly used by the traditional healers to prepare a remedy for ailments. This could be attributed to the additive or synergistic effects that they could have during ailment treatment [30,25]. However, a study conducted in Bolivia by [28] showed that most remedies were prepared from a single medicinal plant species.

Most of the traditional healers were found to have poor knowledge on dosage and antidote while prescribing remedies to their patients and most of the remedies were reported to have no serious adverse effects except vomiting and temporary inflammations. This could be attributed to the low toxicity of the remedy preparations of the medicinal plant species used by the traditional healers in the study area [27].

The reported medicinal plants of the study area were mostly indicated to be abundant and rare. Furthermore, the abundance of medicinal plant species showed significant variation with respect to their marketability, parts used and added values. This significant variation in abundance between non-marketable and marketable medicinal plant species could be attributed to the absence of commercialization pressure, which is believed to reduce over-harvesting of non-marketable medicinal plant species in the study area. On the other hand, the significant difference observed on the abundance of the medicinal plant species with respect to plant parts used and their added values might have resulted due to the various threatening factors mainly the increased anthropogenic pressure on those medicinal plant species of the study area.

The indigenous knowledge among traditional healers with regard to their age and educational level was similar. This might be attributed to equal access of their family members to the existing indigenous knowledge regardless of age and educational level. The proportion of healers who used to transfer their knowledge and those who didn't use to transfer was also similar. This reveals that some of the traditional healers might have given much attention to the indigenous knowledge transfer while others kept the knowledge with them for the sake of secrecy or they might have little concern regarding the value of indigenous knowledge.

According to the available literatures, some of the reported medicinal plant species were found to have some phytochemical and biological activities. Antibacterial activities were reported from the essential oil of *C. anisata *leaves [31]. This validates the reported traditional use of this species by local healers to treat *Naqarsaa *in the current study area. Leaf extracts of *C. hirsuta *was indicated to have strong antifungal activity on certain species of fungi [32]. But this medicinal plant species was reported to treat earache (*Dhukuba Guraa) *by traditional healers of the study area, which needs further study to confirm its antibacterial activity. *In vitro *test by [33] and [34] showed extracts of *C. myricoides *to have antiplasmodial activity. However, in the current study, this species was reported to treat tumor (*Tanacha*). The leaf extracts of *E. abyssinica *showed strong antiviral activity [32] and trypanocidal activity [35] in Rwanda and Uganda, respectively. Antibacterial activity of this species was also reported from East Africa [36] and this agrees with the reported use of this species by the local healers of the study area to treat gonorrhoea (*Dhukuba Dhiraa*). Antiviral activity from methanolic extracts of *P. zeylanica *was reported in Ethiopia [37]. But this species was reported by the traditional healers of the current study area to treat tumor (*Tanachaa*). Root extracts of *Withania somnifera *(L.) Dun showed anticarcinogenic [38,39] and anti-stress activity [40]. The latter activity of this species validates its traditional use by the local healers of the study area to treat evil eye (*Buda*).

## Conclusion

In the present study, twenty seven plant species of medicinal importance were recorded and documented. The majority of the reported medicinal plant species were wild. Many medicinal plant species were also reported to be rare. These demand an urgent attention to conserve such vital resources so as to optimize their use in the primary healthcare system. A rich heritage of indigenous medicinal plant use and knowledge was also recognized. However, awareness creation should be made among the healers so as to avoid erosion of the indigenous knowledge and to ensure its sustainable use and conservation as indigenous knowledge transfer in the study area was oral and some healers were not transferring it all. Further phytochemical and biological activity studies should also be conducted on the reported medicinal plant species of the study area so as to utilize them in drug development.

## Competing interests

The author(s) declare that they have no competing interests.

## Authors' contributions

Both authors contributed equally during the field work, data management and preparation of the manuscript.

## Supplementary Material

Additional File 1 List of medicinal plant species, parts used, methods of preparation, administration and diseases treated. The additional file lists plant family name, scientific name, vernacular name, specimen collection number, diseases treated, plant part used, methods of preparation and route of administration. Click here for file
