# Supplementary material for: Traditional medicinal plant knowledge and use by local healers in Sekoru District, Jimma Zone, Southwestern Ethiopia
Source: J Ethnobiol Ethnomed. 2007 Jun 4;3:24. doi: 10.1186/1746-4269-3-24 (PMC1905946; doi:10.1186/1746-4269-3-24)
Supplement: Additional File 1 — List of medicinal plant species, parts used, methods of preparation, administration and diseases treated. The additional file lists plant family name, scientific name, vernacular name, specimen collection number, diseases treated, plant part used, methods of preparation and route of administration. [file 1746-4269-3-24-S1.pdf]

Additional file 1. List of medicinal plant species, parts used, methods of preparation, administration and diseases treated

Species	Family	Vernacular name	Coll. No	Plant type (Cultivated/ wild)	Disease treated	Part used	Condition of plant used (fresh/dried)	Preparation	Administration	Provenance
Alysicarpus quartinianus A. Rich.	Fabaceae	Hadhaa/ Korcha Hadhaa	Ha & De 15	Wild	*Abiato (shererit)*	Root, leaves	Fresh	Crushed, used alone	Dermal	Ganda Chala
					Amoebiasis	Root	Dried	Powdered, mixed with garlic	Oral	Ganda Chala
					Naqarsaa	Leaves	Fresh	Crushed, Squeezed with water	Oral	Ganda Chala
Calpurnia aurea (Ait.) Benth.	Fabaceae	Ceekaa	Ha & De 39	Wild	Earache (*Dhukuba Guraa*)	Leaves	Fresh	Crushed, squeezed with water	Auricular	Liben
Cassia arereh Del.	Fabaceae	Botoroo	Ha & De 18	Wild	Snake bite	Stem bark	Fresh	Powdered, mixed with tea	Oral	Unquure
Celtis africana Burm. F.	Ulmaceae	Mataqoma (Muka Morma)	Ha & De 28	Wild	*Gnataa (Birid)*	Leaves	Fresh	Squeezed with water	Dermal	Bore
Clausena anisata (Willd.) Hook. f. ex Benth.	Rutaceae	Ulmaye	Ha & De 37	Wild	*Naqarsaa*	Leaves	Fresh	Crushed, Squeezed with water	Oral	Ganda Chala
Clematis hirsuta Perr. & Guill.	Ranunculaceae	Fitii	Ha & De 99	Wild	Earache (*Dhukuba Guraa*)	Leaves	Fresh	Crushed, squeezed with water	Auricular	Ganda Chala
Clerodendrum myricoides (Hochst) R. Br. ex Vatke	Verbenaceae	Marasisa	Ha & De 82	Wild	Tumor (*Tanachaa*)	Root	Fresh	Crushed, Squeezed with water	Oral	Unquure
Croton macrostachyus Del.	Euphorbiaceae	Makanissaa	Ha & De 6	Wild	*Abiato (shererit)*	Leaves	Fresh or dried	Concoction, Pounded, Powdered, squeezed	Dermal	Unquure
					Gnataa (Birid)	Leaves	Fresh	Squeezed with water	Dermal	Liben
Dioscorea praehensilis Benth.	Dioscoreaceae	Wociinoo	Ha & De 31	Cultivated	Tenea versicolor (*Sono*)	Leaves	Fresh	Crushed, used alone	Dermal	Bore
Engleriana woodfordioides (Schweinf.)	Loranthaceae	Harmee	Ha & De 51	Wild	Earache (*Dhukuba Guraa*)	Leaves	Fresh	Crushed, squeezed with water	Auricular	Bore
					Scabies (*Ciitto*)	Root	Fresh	Crushed, mixed with water & vaseline	Dermal	Liben
Entada abyssinica	Fabaceae	Haambaltaa	Ha &	Wild	Gonorrhoea	Root	Fresh	Crushed, squezed	Intrave	Unquure

Steud. ex A. Rich.			De 46		(*Dhukuba Dhiraa*)			with water	nous	e
					Heart failure (*Yelib dikam*)	Stem bark, leaves	Dried	Crushed, powdered, used alone	Oral	Unquure e
Gardenia ternifolia Schumach. & Thonn.	Rubiaceae	Kambeelloo/ Gambeelloo	Ha & De 5	Wild	Haemorrhoids (*Kormomo or Kintaroti*)	Fruit	Fresh or dried	Crushed, powdered, concoction, used alone	Dermal	Liben
Gloriosa superba L.	Colchicaceae	Dawaurahma n/ Rahmaldawa	Ha & De 3	Wild	Tumor (*Tanachaa*)	Root	Dried	Powdered, administered with tea or coffee	Oral	Ganda Chala
					Tumor (*Tanachaa*)	Root	Fresh	Crushed, mixed with water	Oral	Unquure e
					Menustrual pbm (*yalememtat*)	Root	Fresh	Crushed, mixed with sugar	Oral	Bore
Hypoestes triflora (Forssk.) Roem & Schult	Acanthaceae	Togoo	Ha & De 9	Wild/ cultivated	Anaemia (*Hiireenaa Daga*)	Leaves	Fresh	Decoction, mixed with sugar	Oral	Unquure e
Justicia schimperiana (Hochst. Ex nees) T. Anders.	Acanthaceae	Dhumugaa	Ha & De 26	Wild	Gnataa (Birid)	Leaves	Fresh	Squeezed with water	Dermal	Liben
Maesa lanceolata Forssk.	Myrsinaceae	Eija Abbayi	Ha & De 43	Wild	Scabies (*Ciitto*)	Seeds	Dried	Powdered, mixed with edible oil	Dermal	Ganda Chala
Momordica foetida Schumach.	Cucurbitaceae	Hidda Boffaa/Buqee	Ha & De 48	Wild	Gonorrhoea (*Dhukuba Dhiraa*)	Root	Fresh	Crushed, squeezed with water	Intrave nous	Unquure e
					Abdominal distension (*Gara Kassa*)	Leaves	Fresh	Crushed, squeezed, mixed with water	Oral	Liben
Myrica salicifolia Hochst. ex A. Rich.	Myricaceae	Nolee	Ha & De 59	Wild	Earache (*Dhukuba Guraa*)	Leaves	Fresh	Crushed, squeezed with water	Auricul ar	Unquure e
					Naqarsaa	Leaves	Fresh	Crushed, squeezed with water	Oral	Unquure e
Myrsine africana L.	Myrsinaceae	Qachamaa	Ha & De 6	Wild	*Naqarsaa*	Leaves	Fresh	Crushed, Squeezed with water	Oral	Unquure e
Ocimum lamiifolium Hochst. ex Benth.	Lamiaceae	Damakessie	Ha & De 8	Wild	Febrile Illness (*Michii*)	Leaves	Fresh	Pounded, squeezed, used alone	Oral, Dermal	Ganda Chala
Oreosyce africana Hook. f.	Cucurbitaceae	Hiddii	Ha & De 45	Wild	Gonorrhoea (*Dhukuba Dhiraa*)	Root	Fresh	Crushed, squezed with water	Intrave nous	Bore
Plectranthus rupestris	Lamiaceae	Balajuu	Ha &	Wild	Earache	Leaves	Fresh	Crushed, squeezed	Auricul	Bore

Vatke ex Baker			De 47		(*Dhukuba Guraa*)			with water	ar	
Plumbago zeylanica L.	Plumbaginaceae	Umeeraa	Ha & De 11	Wild/ cultivated	Tumor (*Tanachaa*)	Root	Dried	Powdered, mixed with water and sugar	Oral	Bore
					Tumor (*Tanachaa*)	Twig	Fresh or dried	Used alone	Put on the neck as necklac e	Bore
Premna schimperi Engl.	Verbenaceae	Urgessa	Ha & De 4	Wild	*Abiato (shererit)*	Leaves	Fresh or dried	Concoction, Pounded, Powdered, squeezed	Dermal	Liben
					Naqarsaa	Leaves	Fresh	Crushed, mixed with water	Oral	Liben
Tapinanthus globiferus (A. Rich.) Tiegh.	Loranthaceae	Dheertu Makanissaa	Ha & De 98	Wild	Tumor (*Tanachaa*)	Leaves	Fresh	Crushed, mixed with water	Oral	Liben
Vernonia amygdalina Del.	Asteraceae	Ebichaa	Ha & De 30	Wild	*Abiato (shererit)*	Leaves	Fresh or dried	Concoction, Pounded, Powdered, squeezed	Dermal	Bore
Withania somnifera (L.) Dun	Solanaceae	Qorcha Buda (gizawa)	Ha & De 41	Wild/ cultivated	Evil eye (*Buda*)	Leaves	Fresh	Squeezed, used alone	Oral	Liben
